# Case Report: Effectiveness of secukinumab in systemic sclerosis with early skin progress after autologous hematopoietic stem cell transplantation and end-stage kidney disease

**DOI:** 10.3389/fimmu.2023.1294496

**Published:** 2023-11-17

**Authors:** Patrick-Pascal Strunz, Hannah Labinsky, Lea-Kristin Nagler, Jan Portegys, Matthias Froehlich, Michael Gernert, Marc Schmalzing

**Affiliations:** Department of Internal Medicine 2, Rheumatology/Clinical Immunology, University Hospital Würzburg, Würzburg, Germany

**Keywords:** scleroderma, IL-17 inhibition, melphalan, high-dose chemotherapy, renal failure, hemodialysis, stem cell transplantation, renal crisis

## Abstract

Autologous hematopoietic stem cell transplantation (aHSCT) represents an effective treatment option in patients with severe forms of systemic sclerosis (SSc) by resetting the immune system. Nevertheless, secondary autoimmune disorders and progressive disease after aHSCT might necessitate renewed immunosuppressive treatments. This is particularly challenging when organ dysfunction, i.e., end-stage kidney failure, is present. In this case report, we present the unique case of a 43-year-old female patient with rapidly progressive diffuse systemic sclerosis who underwent aHSCT despite end-stage renal failure as consequence of SSc-renal crisis. Therefore, conditioning chemotherapy was performed with melphalan instead of cyclophosphamide with no occurrence of severe adverse events during the aplastic period and thereafter. After aHSCT, early disease progression of the skin occurred and was successfully treated with secukinumab. Thereby, to the best of our knowledge, we report the first case of successful aHSCT in a SSc-patient with end-stage kidney failure and also the first successful use of an IL-17 inhibitor to treat early disease progression after aHSCT.

## 1 Introduction

Since the ASSIST, ASTIS, and SCOT trials, autologous hematopoietic stem cell transplantation (aHSCT) is an established and proven highly effective treatment of progressive and refractory systemic sclerosis (SSc) (1–5). Despite this effective treatment, progressive disease and secondary autoimmune diseases after aHSCT might necessitate renewed immunosuppressive treatments in up to 60% of the cases (6, 7). So far, there are no recommendations for managing this scenario, especially when further challenging aspects like organ dysfunctions, i.e. kidney failure, are present (7).

In Japanese trials, inhibition of the IL-17 pathway has recently been described as an effective and promising new target in treating SSc (8, 9). In this context, we want to report our case of a 43-years old female patient with end stage kidney disease and hemodialysis after renal crisis and early skin progress after aHSCT, effectively treated with the IL-17A inhibitor secukinumab.

## 2 Case presentation

**Figure 1:**
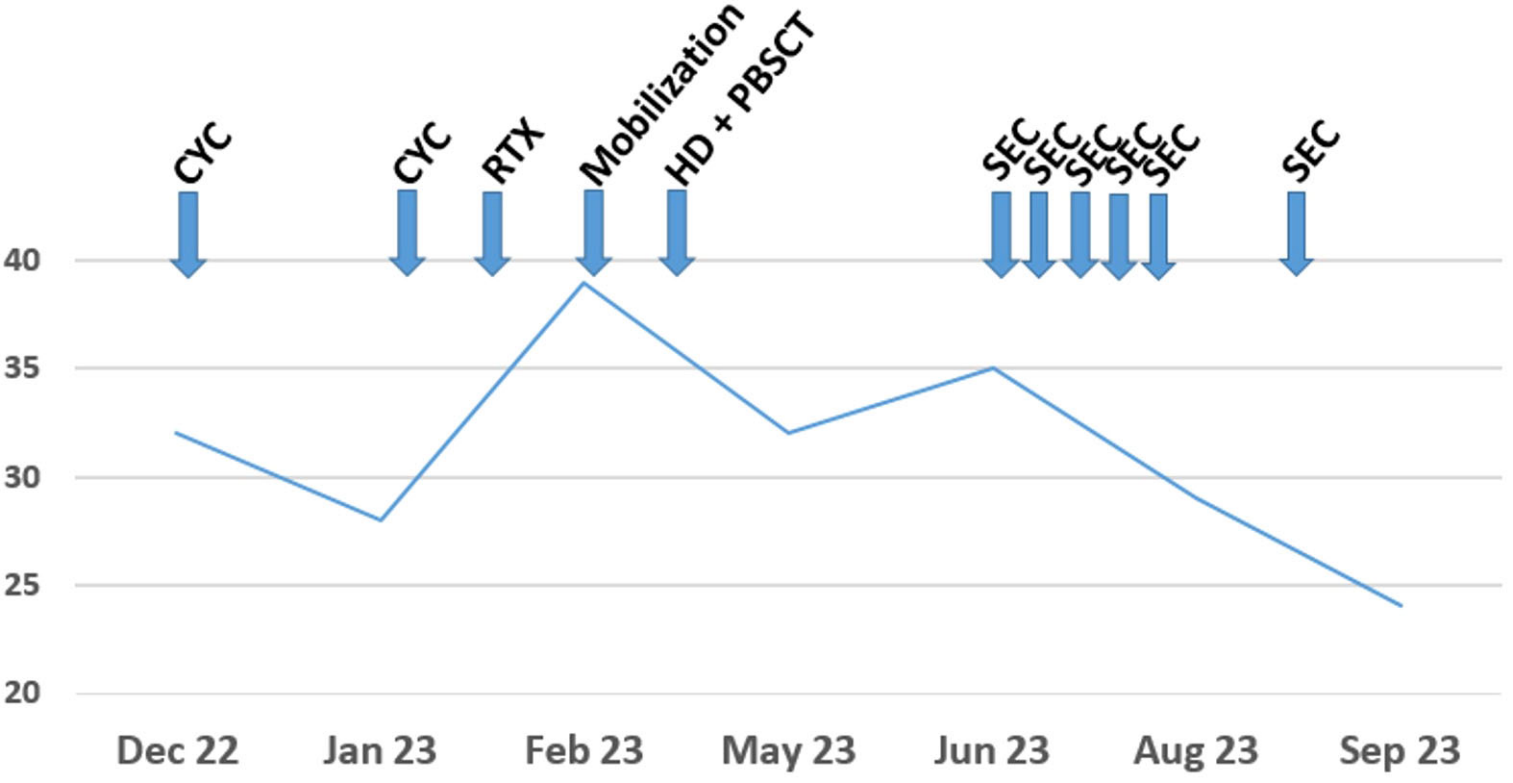
Modified Rodnan skin score in dependence on the treatment regimen from December 2022 until September 2023. Assessment of mRSS was always performed by the same investigator. The extent of the mRSS on the y-axis is shown over time. Early disease progress after aHSCT made treatment with SEC necessary. SEC achieved a rapid and sustained skin response. For the exact values, see **Table 1**. CYC, cyclophosphamide; RTX, rituximab; HD, high dosage chemotherapy; PBSCT, peripheral blood stem cell transplant; SEC, secukinumab.

In December 2022, a 43-year-old female patient with Scl-70 positive and rapidly-progressive SSc was admitted to our clinic for initiation of immunosuppressive treatment. Diagnosis of SSc with pulmonary and cardiac involvement had been established one year before. Initial modified Rodnan skin score (mRSS) was 32, forced vital capacity (FVC) was 79% predicted, and estimated glomerular filtration rate (eGFR, MDRD) was 99 ml/min (see **Table 1**). Assessment of mRSS was routinely performed by the same senior consultant in rheumatology during the case, an experienced investigator of the former ASTIS trial. Due to progressive skin manifestation, treatment with cyclophosphamide (cyc) 750mg/m² was initiated. After two cycles with initial treatment response, a flare with rapid skin progression occurred in January 2023 (see **Figure 1**) associated with stiffness, joint pain and severe impairment in daily activities. In this situation, the indication for aHSCT was set. Rituximab was administered in a dose of 1x 1000mg as a bridging therapy to transplant. Analogous to the ASTIS protocol, mobilization chemotherapy for stem cell apheresis containing cyc in a dose of 2000mg/m² per day on two consecutive days was used followed by stimulation with granulocyte colony-stimulating factor (G-CSF) in February 2023. After successful apheresis of CD34-selected stem cells, the patient was discharged with a slightly reduced eGFR of 72 ml/min.

**Table 1:** Disease parameters between November 2021 (primary diagnosis) and September 2023.

	11/21	12/22	01/23	02/23	03/23	05/23	06/23	08/23	09/23
mRSS	n. d.	32	28	39	n. d.	32	35	29	24
FVC predicted in %	89%	n. d.	79%.	n. d.	n. d.	n. d.	n. d.	79%	n. d.
eGFR (MDRD) in ml/min	80	99	94	104	30	6	dialysis	dialysis	dialysis
Creatinine in mg/dl	0.79	0.69	0.72	0.66	1.94	7.39	dialysis	dialysis	dialysis

n.d, not done; eGFR, estimated glomerular filtration rate.

**Figure 2:**
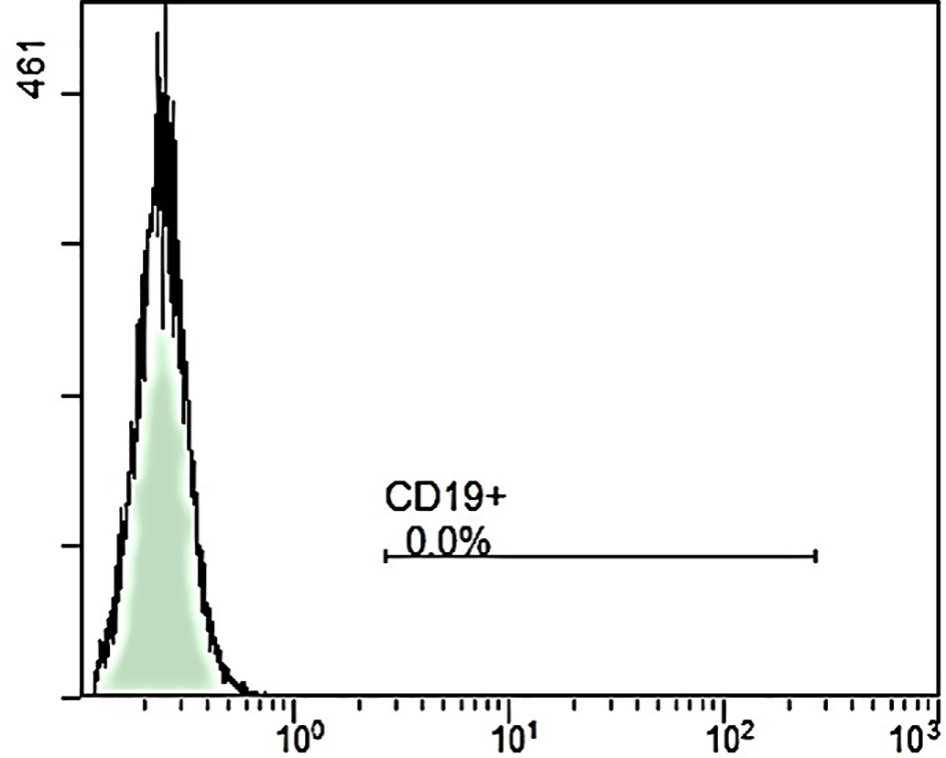
Peripheral blood flow cytometry in June 2023. No B cells were detectable in the peripheral blood in June 2023 when the systemic sclerosis relapsed. At that time, rituximab pre-treatment was only 5 month ago as well as autologous hematopoietic stem cell transplant was only 2 month ago. This finding leads together with the increased risk of severe infectious complications by re-treatment with rituximab right after transplant to the decision against re-treatment with rituximab to address the early skin relapse after autologous hematopoietic stem cell transplant.

At re-admission for aHSCT three weeks later, the patient presented progressive renal failure with an eGFR of 30ml/min, mild hemolysis, 0,7% schistocytes, and severe arterial hypertension (mean blood pressure values of 190/90 mmHg). After the exclusion of other causes, we interpreted this complication as a renal crisis, a known complication of SSc (10, 11). Therefore, we administered high-dose of an ACE inhibitor (captopril) and calcium channel inhibitor (amlodipine) to control blood pressure (10, 11). Due to aggressive and extensive skin involvement (mRSS 39 at that time), we decided to perform high-dosage chemotherapy with aHSCT after an ineffective trial with upadacitinib for two weeks. The JAK inhibitor (JAKi) upadacitinib was given over 2 weeks because the JAKi tofacitinib had been shown to be effective in prior data with respect to SSc skin involvement (12). Upadacitinib was chosen instead of tofacitinib due to severe kidney failure. Regarding the further deterioration of kidney function (eGFR 16 ml/min), cyclophosphamide was replaced by melphalan (50mg/m² on two consecutive days) accompanied by reduced ATG dose (total dosage of 15mg/kg grafalon) for four days as conditioning regimen. Engraftment of leucocytes, defined as ANC>500/µl, took place on day 14 and a total of 7 red blood cell and 4 platelet transfusions were necessary. Besides a mucositis, the aplastic period proceeded without further complication so that the patient could be discharged on day 28 after aHSCT. Unfortunately, kidney function did not improve and hemodialysis became necessary in Mai 2023. After initial response of skin involvement (mRSS declined from 39 to 32 after aHSCT (see **Figure 1**)), SSc started to flare up again with skin tenderness and re-increase of diffuse scleroderma accompanied by reduced range of motion, (mRSS 35) in June 2023. Because the last rituximab infusion was only five month ago as well as aHSCT was only two month ago the peripheral blood flow cytometry consecutively revealed no peripheral B cells (see **Figure 2**). Since the patient showed disease progression after rituximab in the past, re-therapy with rituximab was no promising option in our opinion and other immunosuppressive treatments were contra-indicated due to end-stage kidney failure with the necessity of hemodialysis. Thus, we decided to administrate the IL-17A inhibitor secukinumab (300mg on the days 0, 7, 14, 21, 28 and then monthly), starting in June 2023. At re-assessment in August 2023, the patient presented with significant clinical improvement of the skin (mRSS 29 (**Figure 1**)). Notably in this context, the patient was able to stretch her elbows for the first time since onset of SSc due to the improvement of the skin sclerosis. At the follow up visit in September 2023, the skin thickening had still further improved compared to August with reaching a mRSS score of 24 now. Furthermore, pulmonary involvement remained stable at FVC 79% predicted with no further deterioration (FVC January 2023: 79%). No adverse events due to secukinumab administration have been observed so far.

## 3 Discussion

In this case report, we present the unique case of a 43-year-old female patient with rapidly progressive diffuse cutaneous systemic sclerosis who underwent aHSCT despite end-stage renal failure and was successfully treated with secukinumab for early skin relapse.

In our view, this case is remarkable for several reasons. Firstly, melphalan was used for the conditioning regimen instead of cyclophosphamide or fludarabine or thiotepa due to the patient’s end-stage kidney disease. High dosage myeloablative chemotherapy with melphalan is well established in treating multiple myeloma but is not routinely used in aHSCT in the context of SSc (1, 4, 5, 13). Secondly, to the best of our knowledge, aHSCT for SSc has never been successfully performed in a patient with end-stage kidney failure before. Regarding the use of melphalan in this context, there is some evidence that it can be safely used in high-dosage chemotherapy in patients with end-stage kidney disease in the context of multiple myeloma and amyloidosis (14). To further reduce toxicity, we decided to use half of the standard dose for treating multiple myeloma (100mg/m²) (13). Strikingly, besides a mucosits grade III no severe adverse events were seen during and after aHSCT indicating that high-dosage chemotherapy with melphalan instead of cyc might be a relatively safe treatment option for SSc-patients with end-stage kidney disease undergoing aHSCT, too.

Furthermore, we describe the first case of successful use of IL-17A inhibition in an SSc-patient with progressive skin disease after aHSCT. IL-17 inhibition with brodalumab has recently been described in Japanese trials as an effective treatment option in SSc with achievement of quick and sustained skin response (8, 9). Evidence for the optimal immunosuppressive treatment after aHSCT is generally low and no standardized treatment recommendations are available. Best evidence exists for the use of rituximab in this scenario (7). However, since the CAST trial, rituximab is increasingly used in the conditioning regimen (15). In case of early disease progression after aHSCT in already rituximab pre-treated patients, re-treatment with rituximab might not be a promising strategy, especially, when the last rituximab infusion is less than 6 months ago. In our patient, we therefore decided against the use of rituximab due to her rituximab pre-treatment only five months ago, the lack of response to that, her absent peripheral B cells at the time of disease progression, and the short latency of two months to aHSCT with increased risk of serious infectious complications like progressive multifocal leukoencephalopathy by reinfusion of rituximab. In this scenario, inhibition of the IL-17 pathway might represent an interesting new and promising treatment option. Especially due to the lack of clear significant efficacy of tocilizumab and abatacept on skin involvement in the faSScinate and focuSSed trial and the ASSET trial, respectively, we decided against treatment with tocilizumab or abatacept (16–18). Thus, we made the decision to use the IL-17A inhibitor secukinumab because of the above-mentioned evidence of the efficacy of IL-17A inhibition and the more favorable risk profile of secukinumab with an overall low risk of severe infectious complications (19). Additionally, there are no contraindications for the use of secukinumab in patients with end-stage kidney disease undergoing hemodialysis. In the three-month follow-up period, no significant adverse events could have been observed so far. The blockade of the IL-17 signaling pathway might therefore be considered a promising new and safe therapy option for SSc-patients in the challenging situation of the necessity of an immunosuppressive treatment after aHSCT. Of course, these observations need to be confirmed by further studies to make general recommendations.

## Data availability statement

The raw data supporting the conclusions of this article will be made available by the authors, without undue reservation.

## Ethics statement

Due to the retrospective nature of the study, ethical approval is not needed by German legislation. The studies were conducted in accordance with the local legislation and institutional requirements. The participants provided their written informed consent to participate in this study. Written informed consent was obtained from the individual(s) for the publication of any potentially identifiable images or data included in this article.

## Author contributions

P-PS: Conceptualization, Data curation, Formal Analysis, Investigation, Methodology, Visualization, Writing – original draft, Writing – review & editing. HL: Writing – review & editing. L-KN: Writing – review & editing. JP: Writing – review & editing. MF: Writing – review & editing. MG: Writing – review & editing. MS: Conceptualization, Investigation, Supervision, Writing – original draft, Writing – review & editing.
